# A demonstration of modularity, reuse, reproducibility, portability and scalability for modeling and simulation of cardiac electrophysiology using Kepler Workflows

**DOI:** 10.1371/journal.pcbi.1006856

**Published:** 2019-03-08

**Authors:** Pei-Chi Yang, Shweta Purawat, Pek U. Ieong, Mao-Tsuen Jeng, Kevin R. DeMarco, Igor Vorobyov, Andrew D. McCulloch, Ilkay Altintas, Rommie E. Amaro, Colleen E. Clancy

**Affiliations:** 1 Department of Physiology and Membrane Biology, Department of Pharmacology, School of Medicine, University of California Davis, Davis, California, United States of America; 2 San Diego Supercomputer Center (SDSC), University of California, San Diego, La Jolla, California, United States of America; 3 Department of Chemistry and Biochemistry, National Biomedical Computation Resource, Drug Design Data Resource (D3R), University of California San Diego, La Jolla, California, United States of America; 4 Departments of Bioengineering and Medicine, University of California, San Diego, La Jolla, California, United States of America; University of Washington, UNITED STATES

## Abstract

Multi-scale computational modeling is a major branch of computational biology as evidenced by the US federal interagency Multi-Scale Modeling Consortium and major international projects. It invariably involves specific and detailed sequences of data analysis and simulation, often with multiple tools and datasets, and the community recognizes improved modularity, reuse, reproducibility, portability and scalability as critical unmet needs in this area. Scientific workflows are a well-recognized strategy for addressing these needs in scientific computing. While there are good examples if the use of scientific workflows in bioinformatics, medical informatics, biomedical imaging and data analysis, there are fewer examples in multi-scale computational modeling in general and cardiac electrophysiology in particular. Cardiac electrophysiology simulation is a mature area of multi-scale computational biology that serves as an excellent use case for developing and testing new scientific workflows. In this article, we develop, describe and test a computational workflow that serves as a proof of concept of a platform for the robust integration and implementation of a reusable and reproducible multi-scale cardiac cell and tissue model that is expandable, modular and portable. The workflow described leverages Python and Kepler-Python actor for plotting and pre/post-processing. During all stages of the workflow design, we rely on freely available open-source tools, to make our workflow freely usable by scientists.

## Author summary

We present a computational workflow as a proof of concept for integration and implementation of a reusable and reproducible cardiac multi-scale electrophysiology model that is expandable, modular and portable. This framework enables scientists to create intuitive, user-friendly and flexible end-to-end automated scientific workflows using a graphical user interface. Kepler is an advanced open-source platform that supports multiple models of computation. The underlying workflow engine handles scalability, provenance, reproducibility aspects of the code, performs orchestration of data flow, and automates execution on heterogeneous computing resources. One of the main advantages of workflow utilization is the integration of code written in multiple languages Standardization occurs at the interfaces of the workflow elements and allows for general applications and easy comparison and integration of code from different research groups or even multiple programmers coding in different languages for various purposes from the same group. A workflow driven problem-solving approach enables domain scientists to focus on resolving the core science questions, and delegates the computational and process management burden to the underlying Workflow. The workflow driven approach allows scaling the computational experiment with distributed data-parallel execution on multiple computing platforms, such as, HPC resources, GPU clusters, Cloud etc. The workflow framework tracks software version information along with hardware information to allow users an opportunity to trace any variation in workflow outcome to the system configurations.

## Introduction

Computational modeling and simulation has proven to be a powerful approach to reveal fundamental mechanisms of the cardiac rhythm in both normal and pathological conditions. Recent studies have expanded modeling approaches to the domain of predictive pharmacology, utilizing functional *in silico* approaches to *predict* drug efficacy, screen for drug toxicity, as well as suggest disease-specific therapies [1–11]. Modeling and simulation as an approach has distinct advantages over classical experimental methods, including the potential for high throughput prediction, choice of model complexity best suited for a given problem, and investigation of a range of physiological, pathophysiological and pharmacological parameters. Furthermore, computational modeling and simulation allows for the prediction of overall emergent effects of specific parameter perturbations on the simulated system.

As computational cardiac models have become increasingly accepted as predictive tools, there has been a recent movement towards utilizing them in applied venues, especially in the domain of safety pharmacology [12, 13]. This transition has required a deep and objective assessment of the need for well-defined criteria to allow for the verification, validation, and uncertainty quantification (VVUQ) of models and model predictions [13–15]. In the VVUQ paradigm, *verification* ensures the computational model accurately solves the equations underlying the mathematical model, and that model reproducibility is ensured regardless of implementation environment (i.e. different computing hardware, compilers, and code libraries), *validation* serves as a measure of the extent, to which the model is accurate in representing the quantities of interest (that may be experimental data), and *uncertainty quantification* determines the extent to which the model output is sensitive (or uncertain in response) to variation, error and uncertainty in the model input. In concert with VVUQ considerations, there has been a determined effort to address the overlapping issues of reproducibility, repeatability and replicability across a variety of computational disciplines via the application of standards [16–19] [14, 15, 20, 21].

CellML and related markup languages like SBML have been utilized to provide a standard, software- and programing language-independent description of the model, which can improve consistency and reproducibility of model description and sharing [22]. No single markup language can represent a full cardiac multi-scale model, although the combination of CellML to describe the ionic model, FieldML (http://physiomeproject.org/software/fieldml/about) for describing the field equations and geometry, and SEDML (https://sed-ml.github.io) [23–26] for describing the protocol of the numerical experiment, could in principle be combined to allow a full description.

Other tools have also been developed, such as CellML API or OpenCor that can automatically implement model representations in markup languages [27, 28]. In this way, it is possible to generate whole cell ODE model equations from a language independent CellML description of the model. There are some examples of integrated frameworks (OpenCMISS [29, 30], Chaste [31–34], CARP [35]) that can solve multi-scale models that are derived from standardized model descriptions and indeed, Chaste and CARP can both be integrated and utilized in Kepler workflows [36]. Some multi-scale simulations do, however, require the use of a variety of solvers and data sets.

Moreover, reproducibility also requires development of standards for simulation and model implementation [20, 23, 25, 26, 37, 38]. SED-ML is a community effort to standardize modeling protocols, but standardized protocols that integrate or connect multiple models represented in standardized model descriptions either requires customized software or a workflow framework [24, 25, 39, 40]. To date, there are a few tools that support SED-ML (Tellurium, JWS Online, SBW Simulation Tool, CellDesigner, COPASI, iBioSim, bioUML, SED-ED) for a limited number of application domains. We tested here whether a workflow platform such as Kepler could provide a reproducible approach for integrating multi-scale models requiring more than one solver, a reproducible protocol for numerical experimentation and provenance tracking. Indeed, none of the tools described are mutually exclusive and workflows such as the one described in this study can be readily expanded to allow inclusion of code generation from CellML, FieldML and SEDML descriptions [16].

In this study, after careful analysis, we decided to utilize the Kepler scientific workflow management system. This framework enables scientists to create intuitive, user-friendly and flexible end-to-end automated scientific workflows using a graphical user interface. Kepler is an advanced open-source platform that supports multiple models of computation [41, 42]. The underlying workflow engine handles scalability, provenance, reproducibility aspects of the code, performs orchestration of data flow, and automates execution on heterogeneous computing resources. A workflow driven problem-solving approach enables domain scientists to focus on resolving the core science questions, and delegates the computational and process management burden to the underlying Kepler Workflow system [43–46]. Further, scientists can parameterize the workflow and perform large-scale search for optimal values in the parameter space. Leveraging the benefits of a workflow driven approach allows scaling the computational experiment with distributed data-parallel execution on multiple computing platforms, such as, HPC resources, GPU clusters, Cloud etc. The framework gives users flexibility to execute the workflows from command-line or GUI. Due to its large open-source developer community, Kepler has a rich library that contains over 350 ready-to-use processing components called 'actors' that can be easily customized.

There have been a number of developments aimed at solving the specific problems of reproducibility, repeatability and replicability. In the context of the work presented here, ‘reproducibility’ refers to zero-difference in outcome between two executions (say W1 and W2) of same workflow (W), when both executions of our workflow W have exact same hardware (H), same software (S), and same initial conditions (P). The Kepler workflow system captures provenance during each execution at multiple levels. The workflow records the workflow parameters, workflow outputs, intermediate data tokens and extracts the hardware system (CPU Cores, Cache, Memory etc.) profile as well. All of this information is recorded in the workflow provenance database. The information includes versions of all the programs. The key components recorded are the version information of the operating system, source code compiler, Python, Kepler, Java and associated source code. The workflow stores this information in Kepler provenance database. The detailed capture of hardware and software environment information enables users to completely reproduce and replicate the results. Our aim is to facilitate the user to setup same initial conditions and hardware environment (if required), and reproduce results in similar fashion. Notably, the definition we use for reproducibility has been described as replicability and repeatability in other descriptions, whereas reproducibility has used to describe an independent reconstruction of the model from the model equations and initial conditions [47–49]. Indeed, despite attempts to develop standard definitions, there is, as yet, no full consensus on the definitions of each term [50].

One of the main advantages of utilization of workflows is that they can integrate code written in multiple languages, allow for variation in application of compilers and can pass information from one code to another. The standardization occurs at the interfaces of the workflow elements (actors) and allows for very general applications and easy comparison and integration of code from different research groups or even multiple programmers coding in different languages for various purposes from the same group.

Kepler workflow elements can be optimized to run on different platforms and compare results (verification), switch code for different models or implementations of the same model and compare results (validation) or run code multiple times with different initial parameters and estimate variation (uncertainty quantification). Also for the reasons above, Kepler workflows are ideally suited for multi-scale modeling due to ability to integrate very different pieces of codes into a workflow and easily parsing input and output parameters between them. Another advantage is that Kepler workflows are easily accessible for non-experts in computational modeling as programming as a detailed knowledge of model inner workings are not needed to run simulations and modify parameters to suit the requirements of the end user.

Here, we present a multi-scale model of cardiac electrophysiology that is executed in the freely available Kepler scientific workflow system [41, 44]. The workflow we present here is a first required step in VVQU by ensuring reproducibility of models through inclusion of provenance information that describes the origin of the model components, referencing to the data, information about any modifications and the associated rationale, as well as the specific components and parameter settings used in each run. We implemented differential equation models of cardiac physiology that automate the execution of simulations with user defined options of outputs from a single cell (0-dimensional), 1 or 2-dimensional tissue, and a pseudo-ECG output, which can be compared to experimental or clinical data.

Many instances of models can be used with varying input parameters, and the models can be linked in the workflows in various ways. For example, single cell models can be linked to an idealized 1-dimensional fiber model, which allows us to compute a signal averaged pseudo ECG that captures temporal and spatial electrical potential gradients of a propagating wave. Another example demonstrates a thousand instances of the single cell model being linked to a 3-dimensional transmural wedge preparation for investigation of ectopic sources. In addition to these multi-modal choices, the framework can also be reused for multispecies comparisons. Users can control a wide range of input parameters from a simplified command-line, or GUI interface. The workflow is portable and scalable, having the flexibility to run on any platform a user chooses: local workstations, small clusters, or remote HPC resources.

The computational workflow we present here represents a proof of concept of a platform for the robust integration and implementation of a reusable and reproducible cardiac cell and tissue model that is expandable, modular and portable. The detailed checkpointing of version information along with hardware information gives users an opportunity to trace any variation in workflow outcome to the system configurations, when the infrastructure cannot be exactly replicated. In addition to storing in the database, the workflow generates an execution report for each workflow execution that includes the important workflow parameters, input information, software version and hardware system profile.

## Methods

### Methodological overview of the workflow

**Fig 1:**
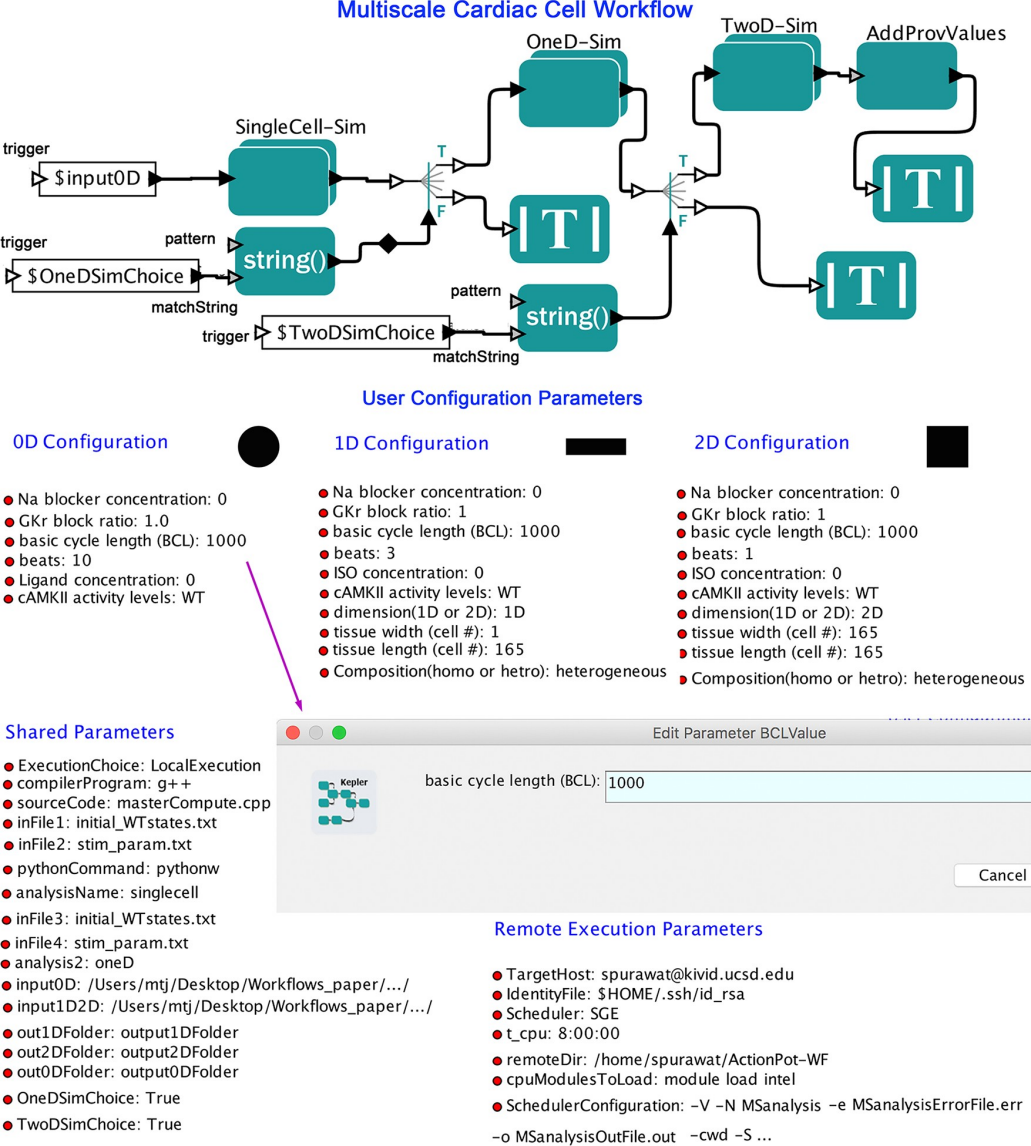
Multiscale cardiac cell workflow. The main interface workflow contains single-cell or zero-dimension (SingleCell-Sim—black circle symbol), one-dimension (OneD-Sim—black rectangle symbol), and two-dimension (TwoD-Sim—black square symbol) tissue simulation modules, as well as user configuration parameters.

A cardiac ventricular electrophysiology modeling and simulation use case:

We present an automated computational workflow (**Fig 1**) that can perform simulations to generate user defined instances and configurations of a single-cell cardiac action potential, conduction of a cardiac action potential in a 1-dimensional (1D) or 2-dimensional (2D) tissue representation and generation of a signal average of electrical activity in time and space to represent a pseudo-ECG.

Please access all codes and associated files and attributes via the GitHub link below. The repository contains specific instructions for use of Kepler System with new source codes. The user-manual provides detailed outlines of how to install Kepler, modify workflow parameters, choose the execution platform and get results from the multi-scale cardiac workflow. The user manual can be accessed at the root of the git repository under filename: “UserManual.docx”

https://github.com/ClancyLabUCD/Workflow_Kepler

Here we demonstrate several example scenarios including: (a) Deployment of the workflow for a single-cell simulation to predict a cardiac action potential with a defined set of input parameters, (b) a configuration for a 1-dimensional cardiac tissue simulation, or (c) a 2-dimensional cardiac tissue simulation.

The model formulations for ventricular cells (the Soltis-Saucerman model [51], Morotti-Grandi model [52], or Grandi-Bers model [53] merged with the Soltis-Saucerman model) were implemented in the Kepler workflow. The source code of simulation models has been implemented in C++ and is compiled during the workflow execution using icc or gcc compiler, depending on the execution platform and compiler availability. Users can use the source code provided by us or attach their custom developed simulation models by editing the workflow parameter “sourceCode.” The workflow gives users a choice to select “compilerProgram” parameter. The workflow integrates multistep single-cell (black circle symbol), 1-dimensional (black rectangle symbol) and 2-dimensional (black square symbol) tissue model simulations in a single automated process (**Fig 1**). The SingleCell-Sim module includes a sub-workflow that performs single-cell simulation. Likewise, OneD-Sim and TwoD-Sim modules perform 1-dimensional and 2-dimensional tissue model simulations, respectively. The workflow includes user configuration components, simulation components with multiple execution choices and post-processing components for each model.

**Fig 2:**
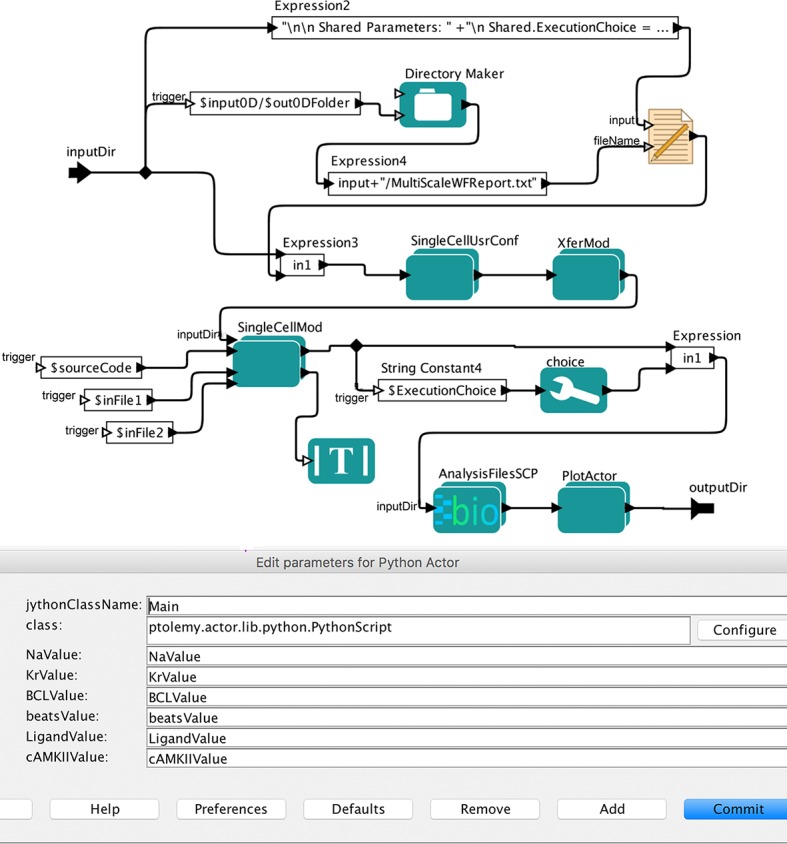
User configuration module. The module “SingleCellUsrConf” is the User Configuration Module for single-cell simulations (SingleCell-Sim, see Fig 1). We created the user configuration module to allow users to control simulation constraints such as Na^+^ and rapid delayed rectifier K^+^ channel (*I*_Kr_) blocker concentrations, ligand (β-blocker isoproterenol) concentration, CaMKII activity levels, number of beats, basic cycle length (BCL) i.e. heartbeat duration and other controls for single-cell simulation through workflow parameters.

User configured parameter settings and initial conditions also allow the end user to control simulation constraints for single-cell, 1D and 2D modules (such as Na^+^-blocker concentration; rapid delayed rectifier potassium channel conductance, *G*_Kr_, block ratio; ligand (β-blocker isoproterenol) concentration, CaMKII (Ca^2+^/calmodulin-dependent protein kinase II) activity levels; number of beats and others through workflow parameters. The simulation constraints are ported as workflow parameters, which can be modified and passed to the simulation models using the user configuration module. This workflow module is implemented using Kepler Python actor and Python libraries. Users can seamlessly configure the simulation parameters simply by changing workflow parameter values through command line or GUI as shown in **Fig 1** (purple arrow). Many instances of these models can be used with varying input parameters, and the models can be linked in the workflows in various ways. The internal structure of the workflow element (actor) is shown in **Fig 2**. User parameter configurations can also be expanded to include more parameters by modifying a workflow actor.

### Multiple execution choices

**Fig 3:**
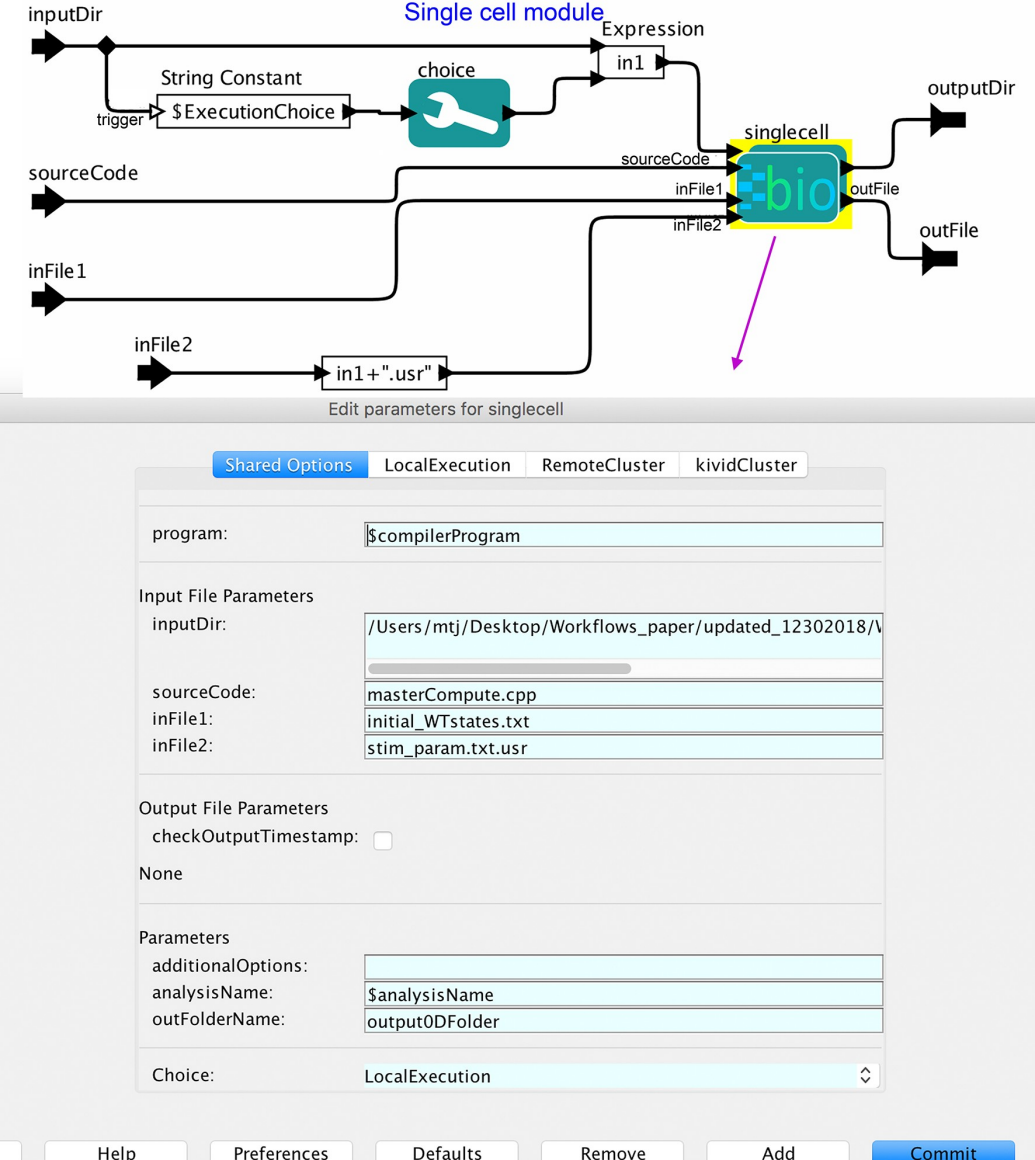
Multiple execution module. The “SingleCellMod” module is the Core Simulation Module for single-cell simulations (SingleCell-Sim, see Fig 1). The Kepler’s Execution Choice actor was created to provide user options of multiple computing platforms based on the use case, data size and the resource availability.

The workflow incorporates flexibility for the end-user’s choice of platform depending on the use case and resource availability. Users can run the workflow on multiple computing platforms such as local, private clusters, and remote HPC clusters by configuring execution choice parameters for individual processes. Kepler allows customization of each execution instance of a workflow with user input parameters. In **Fig 3**, the Kepler’s Execution Choice actor was created in the Core single-cell module. The Local Execution Options and the Remote execution options are also available in the options menu at the top of the GUI. The capability of multiple execution choice on different hardware platforms is achieved by using the Kepler workflow system. By design, the Kepler framework is capable of automatically creating new jobs for execution. This functionality enables scientists to change execution platforms (local or remote) without any additional user scripting.

### Post-processing and visualization module

The post-processing module generates output data files from single-cell, 1D and 2D tissue simulation results. The workflow uses Python libraries and Kepler actors to post process the simulation results and generate plots for simulated action potentials (AP), main ionic currents (*I*_Ca,_ *I*_Kr_, *I*_K1_, *I*_NCX_, *I*_Na_, *I*_to_, *I*_Ks_), intracellular (cytosolic) and sarcoplasmic reticulum concentrations of Ca^2+^ and Na^+^ in single cells, pseudo ECG in a 1D-simulation, and snapshots of AP propagation in 2D tissue.

Further, the Kepler workflow automates the provenance collection, execution report generation and reproducibility. For basic execution of the workflow in “as-is” condition, users do not need expertise in the technologies used, and can execute the workflow using GUI and command line.

### Methods for cardiac simulations executed in this study

All simulations of three cardiac myocyte models (the Soltis-Saucerman model [54], Morotti-Grandi model [52], or Grandi-Bers model [53] merged with the Soltis-Saucerman model) were encoded in C/C++, and run using GCC complier on Mac Pro or Linux computers.

The numerical method used for updating the voltage was forward Euler. Single cell action potentials (APs) and selected ionic currents were recorded. For higher dimension simulations, we simulated a transmural fiber composed of 165 ventricular cells (Δx = Δy = 100 μm) connected by resistances to simulate gap junctions [55]. The transmural fiber contains an endocardial region and epicardial region with a linear decreased in APD as indicated by experimental data [56, 57]. G_Kr_ was used as the index value of endocardium in the cell #1, and the index value of epicardium in cell #165. We can simulate a heterogeneous **2D** cardiac tissue composed of 165 by 165 cells with Δx = Δy = 100 μm. The tissue contains an endocardial region and epicardial region with a linear decreased in APD as indicated by experimental data [56, 57]. Channel conductance and gap-junction parameters are same as in the one-dimensional simulations. Current flow is described by the following equation:

$$\frac{\partial V(x,y,t)}{\partial t}=D_{x}\frac{\partial^{2}V(x,y,t)}{\partial x^{2}}+D_{y}\frac{\partial^{2}V(x,y,t)}{\partial y^{2}}-\frac{I_{ion}-I_{stim}}{C_{m}}$$

Where V is the membrane potential, x and y are distances in the longitudinal and transverse directions, respectively, D_x_ and D_y_ are diffusion coefficients in the x and y directions, C_m_ is membrane capacitance (C_m_ = 1). I_stim_ is 180 mA/cm^2^ for the first 0.5 ms. We also incorporated anisotropic effects by setting D_x_ and D_y_ such that the ratio of conduction velocities is 1:2 [58].

#### Pseudo-ECG computation

Extracellular unipolar potentials (Φ_e_) generated by the fiber in an extensive medium of conductivity σ_e_, were computed from the transmembrane potential V_m_ using the integral expression as in Gima and Rudy [59]:

$$\Phi_{e}(x^{\prime })=\frac{a^{2}\sigma_{i}}{4\sigma_{e}}\int (-\nabla V_{m})\cdot [\nabla \frac{1}{r}]dx$$

$$r=[{\left(x-x^{\prime }\right)}^{2}+{\left(y-y^{\prime }\right)}^{2}{]}^{1/2}$$

Numerical results were visualized using Matplotlib from Python. The workflow requires users to install Kepler 2.5, bioKepler 1.2, Matplotlib, GCC version 4.2.1. Please see Action Potential Workflow User Manual for details.

## Results

### Modeling and simulation in the workflow

One of the key added advantages of using Kepler Workflow system is the ability to deploy new source code easily. To facilitate execution of new cardiac cell models, the path to C++ source code file is parametrized in the Kepler Workflow. If a scientist wants to use their customized cardiac cell model, she/he can edit the Kepler Workflow parameter called 'sourceCode' under the category of 'SharedParameters', to point to the directory where the desired C++ source code resides. Further, the parameters unique to a given source code can be defined in a file called ‘stim_param.txt’. The last step in parametrization is to add a placeholder in the Kepler user interface using the ‘Parameter’ option under the ‘Workflow Input’ menu.

**Fig 4:**
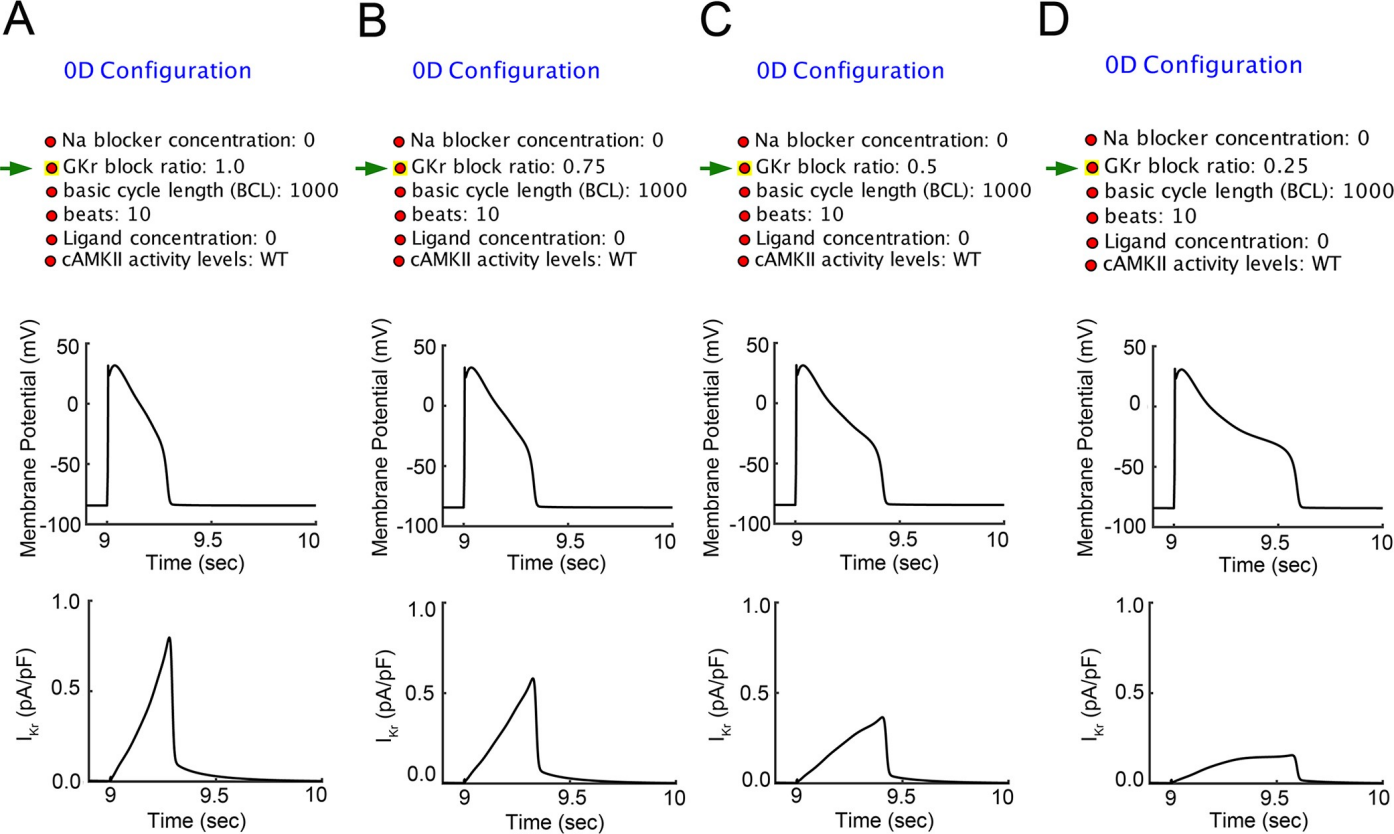
Simulated human ventricular single-cell model via kepler workflow with or without *I*_Kr_ reduction. **A.** User configurations are shown in the left panels. Simulated single-cell action potential (AP–middle panel), and the time course of original *I*_Kr_ (control) during the AP (bottom). **B—D** Simulated single-cell action potentials (middle) with reduced *I*_Kr_ (bottom). *G*_Kr_ block ratio was set to 0.75 (B), 0.5 (C) and 0.25 (D) in 0D configuration setting (green arrows–left panels), corresponding to 25, 50 or 75% *I*_Kr_ block, respectively.

We first demonstrate the potential for the Kepler workflow environment to be used to run batch simulations for a simulated human ventricular single-cell model for varying degrees of *I*_Kr_ reduction (**Fig 4**). The workflow allows users vary rapid delayed rectifier potassium channel conductance, *G*_Kr_, in the simulations. **Fig 4** illustrates single-cell APs and the time-course of *I*_Kr_ through the Kepler workflow with varying *G*_Kr_. In the top of panels **A-D**, various end user configurations for input parameters are shown for each simulation instance. In the middle row, simulated single-cell action potentials are shown. In the bottom row, the time-course of *I*_kr_ during the AP is shown. The *G*_kr_ was reduced via the indicated (green arrows–top panels) block ratios of 1 (used as control, panel **A**), 0.75 (**B**), 0.50 (**C**) and 0.25 (**D**), corresponding to *I*_Kr_ block of 0, 25, 50 and 75%, respectively.

**Fig 5:**
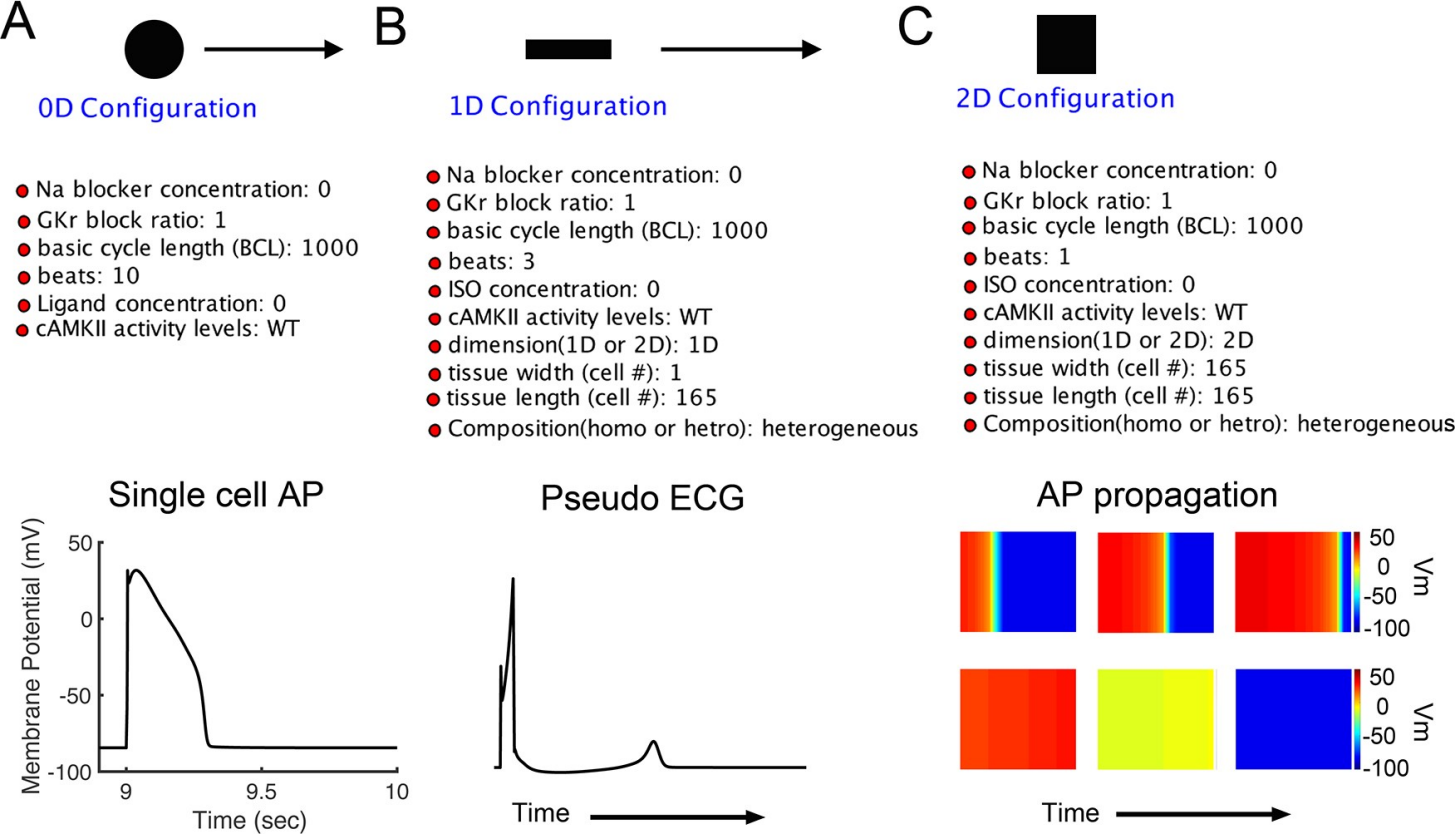
The workflow integrates multistep 0D (single-cell), 1D and 2D model simulations in a single automated process. **A.** Simulated results of single-cell action potential (bottom panel). **B**. The pseudo ECG (bottom panel), and **C**. Six distinct time snapshots of 2D action potential wave propagation (bottom panel) from endocardial region (left) to epicardial region (right), with a linear decrease in APDs.

In **Fig 5**, we demonstrate expansion of the workflow beyond single-cell simulation to user defined 1D and 2D-simulations. The workflow generates a single cell cardiac ventricular action potential (**Fig 5A)**, as well as a one-dimensional simulation and a pseudo-ECG (**Fig 5B**) and then ingests steady-state results from 1D simulations to seed 2D simulations shown in **Fig 5C**. In this example, the single cell was simulated at a pacing rate of 1 Hz and 10 action potentials were generated. The last AP (10^th^ beat) is shown in **Fig 5A** (bottom panel). In the tissue simulations, we simulated a heterogeneous fiber (with a linear decrease in AP duration from endocardial to epicardial region [57] (i.e. from the innermost to the outer layer of the cardiac tissue) composed of 165 ventricular cells (parameter tissue length = 165 cells in **Fig 5B**, top) for three beats. The pseudo-ECG is shown in **Fig 5B** (bottom panel). In the panel C, we demonstrated 2D AP wave propagation in response to one stimulus (a planar wave). The workflow simulated a heterogeneous 2D cardiac tissue composed of an array of 165 cells by 165 cells (1.65 cm x 1.65 cm) [57].

**Fig 6:**
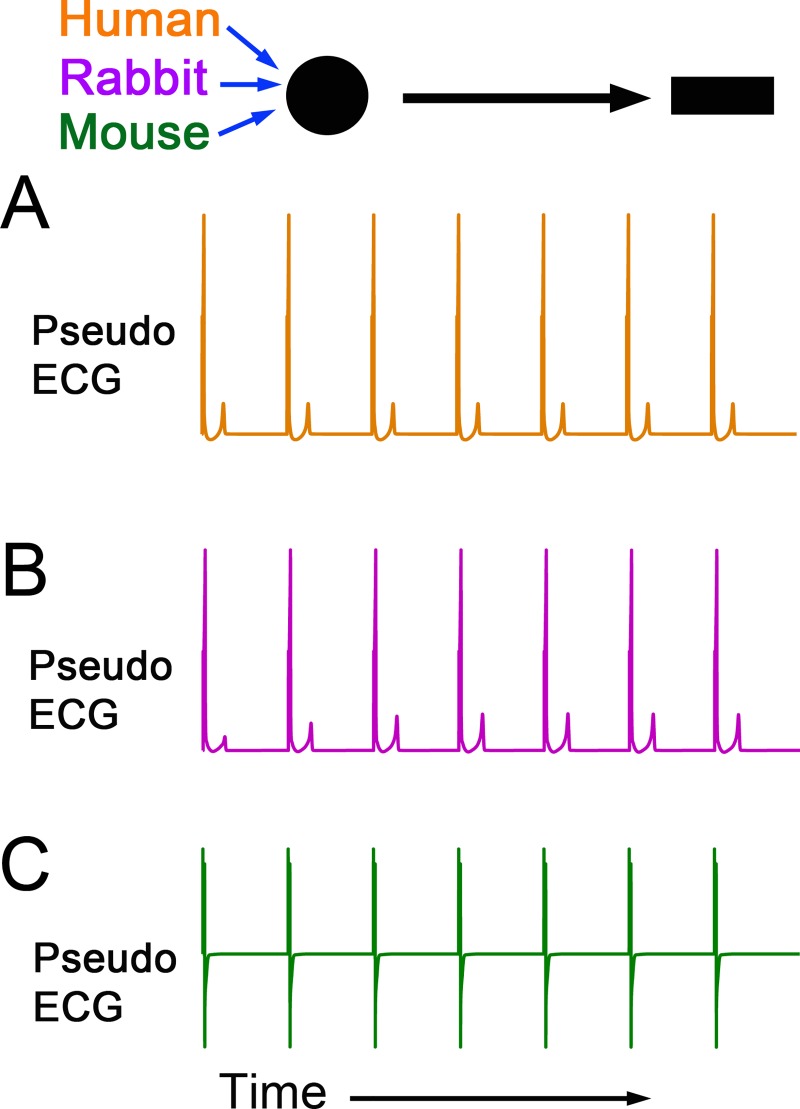
Simulated multispecies ventricular single-cell (0D) and 1D simulations depending on user choice. The multiscale cardiac cell modeling workflow presents from 0-Dimension (circle) to 1-Dimension (rectangle). **A—C.** Pseudo-ECGs were computed in the Human (orange), Rabbit (purple) and Mouse (green) ventricular cell models.

In the example shown in **Fig 6**, we tested three different species models and performed simulations to generate propagation of an action potential in one dimension using the topology shown in **Fig 6 (top)**. Pseudo-ECGs are shown in response to seven stimuli at 1 Hz in Human (**Fig 6A—orange**), Rabbit (**Fig 6B—purple**) and Mouse (**Fig 6C—green**). This example demonstrates how the workflow cyberinfrastructure can also be re-used as a multi-species simulator by utilizing single cell cardiac ventricular computer models as inputs into the higher dimensional models. The cell model of choice can be linked to an idealized one-dimensional fiber model, which can be used to compute signal averaged pseudo ECG traces (**Fig 6A–6C**). They capture temporal and spatial gradients of electric potential during a simulation that tracks conduction and repolarization of a propagating wave.

## Discussion

There has been a tremendous increase in both the number of cardiac models in existence, and in model complexity over the last several decades, correlating with both an increase in computational power, and dramatically reduced computational cost. These developments have created the potential for cardiac cell models and their mathematical and/or agent-based model components to be reused and coupled with one another, creating flexible, modular, portable and potentially scalable models that can account for a range of attributes [60]. The potential for linking models together in new ways also suggests construction of multi-scale models from existing models at various temporal and spatial scales.

To ideally enable model modularity, reuse, reproducibility, portability and scalability, a model execution platform should be able to provide the reuse of code, reproduction of reported *in silico* predictions, as well as a way to run simulations in an efficient, expandable, modular and portable manner. Scientific workflow tools allow exactly these elements and can provide a user interface and potential for automation and optimization of software and hardware elements of the model execution. Workflows derive from the concept of directed graphs with individual nodes that represent discrete computational components that can be optimized to execute on distinct hardware architecture [61–64]. A scientific workflow is conceptualized as a set of tasks performed on a collection of datasets. The workflow-based design enables scientists to break large computational tasks into smaller manageable and reusable modules (nodes). The data flows through these modules (nodes) and gets transformed. The scientists can collaborate effectively on a large-scale problem by bringing their expertise to different modules in a workflow. Data and results flow between the individual nodes.

The computational overhead involved with workflow implementation is during the start of the Kepler Workflow Engine, and the added cost of building the workflow graph in the Kepler GUI. However, this is a one-time cost during a single execution. Once the Kepler Workflow Engine is up and running, the real advantage comes from automated execution, automated provenance collection, and parameterization driven extensibility benefits. In essence, the end user will get these benefits at least, and these can be enhanced by creation of a wrapper mechanism using the Kepler system, that enhances modularity, shareability and extensibility of their work. Wrapping with Kepler Workflow system enhances the portability of source code—it can work on local machine, or on a distributed cluster—so users are not required to modify or write any script for change in execution platforms. Moreover, diversity of parameters can be handled at two levels, when designing the wrapper workflow. The parameters common to an application area, can be abstracted away and customized at the workflow level (indicated by purple arrow in the Fig 1). The parameters unique to a given source code can be defined in a file called ‘stim_param.txt’ and the user needs to add a placeholder (Fig 1 –User configuration Parameters) in the workflow definition using the ‘Parameter’ option under the ‘Workflow Input’ menu.

The added cost of Kepler workflow system can be hedged by exploiting potential to parallelize codes across distributed systems, for problems involving large-scale computation and large datasets. The Kepler system has inbuilt mechanisms to quickly divide and conquer large computations in batch parallel computations. For cases involving specific cost benefit analysis, since the computational overhead of Kepler is dependent on each workflow, we suggest performing case-specific measurement of ‘Kepler + SourceCode + Parallelization Director’ against isolated run of ‘SourceCode’. We are happy to provide support for such efforts, using our support team for open source users of Kepler.

One critical feature of the Kepler that was decisive in selecting this engine, is the “provenance module”. This module archives workflow execution history, parameters, software and hardware signatures. Workflows Provenance can help preserve evidence and data from experiments to achieve reproducibility [43, 65, 66]. The Kepler reporting module generates informative and detailed summaries of the execution that include user configuration parameters used during the execution in various simulation steps, version of respective software tools, and system hardware information on which the workflow is executed. This “execution-signature” can drastically reduce time required to write reports or methods and material section in scientific publications, enabling domain experts to focus their energy on problem solving [43, 45, 65, 66]. Use of Kepler enabled us to delegate these critical components to the framework, and allowed us to focus on the science behind the problem.

It is important to note that workflow frameworks are not an alternative to markup languages for model description or simulation experimentation or an alternative to specialized packages that can integrate more than one kind or scale of model, but rather than efficient and reproducible approach to multi-scale modeling using multiple component models, software tools and data sets that facilitates usability, sharing and provenance tracking.

Here, we demonstrated the application of the freely-available Kepler scientific workflow system to execute a multi-scale model of cardiac electrophysiology. The workflow allows for modularity, scalability and flexibility in a deployable framework that can be configured by the end-user for maximum flexibility. Like most computational scientists, we have long shared concerns about the reproducibility and reuse of models. Versioning and provenance information can be included in Kepler workflow approach as well as the origin of the model components and user defined components and parameter settings used in each run. In this demonstration, we utilized Kepler to develop a workflow containing differential equation models of cardiac physiology that automate the execution of simulations with user defined options of outputs from a single cell (0-dimensional), 1 or 2-dimensional tissue, and a pseudo-ECG output, which can be compared to experimental or clinical data.

The workflow as presented could be readily adopted and expanded for applied use in the safety pharmacology domain. In both clinical and experimental settings, prolongation of the QT interval of the ECG and related proarrhythmia have been so strongly associated, that a prolonged QT interval is largely accepted as surrogate marker for proarrhythmia. Here we demonstrate how the workflow can be applied to an investigation of the impact of perturbation of the key repolarizing potassium current in the heart, the rapidly activating component of the delayed rectifier potassium current, *I*_Kr_. Mutations in the potassium channel gene encoding *I*_Kr_ or drug-induced inhibition of *I*_Kr_ can lead to inherited or acquired long QT syndrome. The QT interval is a phase of the cardiac cycle that corresponds to action potential duration (APD) including cellular repolarization (T-wave). Our single-cell examples demonstrate that reduction of *I*_kr_ caused AP prolongation (**Fig 4**). In **Fig 5**, the workflow can be used to predict QT intervals in the setting of 1-dimensional tissue or further investigate repolarization phases on 2D AP propagation maps by modifying *I*_Kr_. Finally, in **Fig 6** our Kepler workflows allow to easily demonstrate that cardiac electrical signal propagation varies in different species used in experimental studies. And using this approach we can relate findings from animal model studies and correlate them to clinical human studies as well.

While considerable attention has been given to the prospects of computational modelling and simulation as a platform for prediction of cardiac drug safety, electro-toxicity and proarrhythmia risk assessment, less scrutiny over the choice of model and the impact of model choice on predicted effects has been given. Here we also show how the Kepler multi-scale workflow can be applied to multispecies to allow users to perform preliminary assessments in models for which predetermined selections of validation experiments can be performed.

The Kepler cyberinfrastructure enables biomedical scientists to (1) understand and catalog accuracy for assembly and linking of models through rigorous uncertainty quantification (UQ) and sensitivity analysis, (2) define a common practice and methodology for linking together (big) data and high-throughput, multi-spatial, multi-temporal, and complex models through reusable workflow definitions, execution, and tools, (3) develop a user interface building toolkit, and (4) develop new methods for deployment and distribution of highly scalable, portable, expandable and robust software and platforms. An additional benefit of this approach is that it allows for individual workflow elements to be optimized for hardware to maximize efficient parallel computing. Various processes of the workflow can be distributed to execute on optimized systems and then pass data though linkage between the workflow elements.

In the near future, our next steps will include the development of an online training course package with lecture material, videos and hands-on on this Multi-scale Cardiac Workflow tool on the e-learning platform called Biomedical Big Data Training and Collaborative (BBDTC) as our educational and community outreach efforts. The BBDTC (https://biobigdata.ucsd.edu) is a community-oriented platform that encourages collaborative efforts on training and education to ensure high-quality knowledge dissemination to biomedical big data scientific community. The BBDTC provides easy and intuitive interface to create, launch and share open training materials and tools for biomedical community [42, 67].

Future plans also include goals to integrate this workflow with our Machine Learning based performance prediction module to efficiently schedule different components of the workflow on available computing hardware in a way to gain performance and resource optimization [68–70]. We will couple the workflow with our provenance-based fault tolerance framework to automatically detect failure point and re-start the execution of the workflow from point of failure to save time and resources [71].

In summary, we have developed a Kepler based workflow for multi-scale cardiac electrophysiology that can be utilized and expanded for any number of predictions as defined by the end user. The approach brings us closer to the increasingly shared goal of computational scientists to enable model modularity, reuse, reproducibility, portability and scalability. The workflow concept also allows a model execution platform that allows the reuse of code, reproduction of reported *in silico* predictions, as well as a way to run simulations in an efficient, expandable, modular and portable manner. We have demonstrated an application of the approach by linking models together for construction of multispecies multiscale models from existing models at various temporal and spatial scales.
